# *Leishmania infantum* Genetic Diversity and* Lutzomyia longipalpis* Mitochondrial Haplotypes in Brazil

**DOI:** 10.1155/2016/9249217

**Published:** 2016-03-29

**Authors:** Paulo Eduardo Martins Ribolla, Letícia Tsieme Gushi, Maria do Socorro Pires e Cruz, Carlos Henrique Nery Costa, Dorcas Lamounier Costa, Manoel Sebastião da Costa Lima Júnior, Maria Elizabeth Moraes Cavalheiros Dorval, Alessandra Gutierrez de Oliveira, Mirella Ferreira da Cunha Santos, Vera Lúcia Fonseca Camargo-Neves, Carlos Magno Castello Branco Fortaleza, Diego Peres Alonso

**Affiliations:** ^1^Departamento de Parasitologia, Universidade Estadual Paulista “Júlio de Mesquita Filho” (UNESP), Instituto de Biotecnologia de Botucatu (IBTEC), 18607-440 Botucatu, SP, Brazil; ^2^Departamento de Morfofisiologia Veterinária Centro de Ciências Agrárias, Universidade Federal do Piauí, 64049-550 Teresina, PI, Brazil; ^3^Laboratório de Pesquisa em Leishmaniose Visceral, Instituto de Doenças Tropicais Natan Portella, 64001-450 Teresina, PI, Brazil; ^4^Centro de Ciências Biológicas e da Saúde, Universidade Federal do Mato Grosso do Sul (UFMS), 79070-900 Campo Grande, MS, Brazil; ^5^Programa de Pós-Graduação em Doenças Infecciosas e Parasitárias, Faculdade de Medicina (FAMED), Universidade Federal de Mato Grosso do Sul (UFMS), 79070-900 Campo Grande, MS, Brazil; ^6^Superintendência de Controle de Endemias (SUCEN), 01027-000 São Paulo, SP, Brazil; ^7^Departamento de Doenças Tropicais, Faculdade de Medicina de Botucatu, Universidade Estadual Paulista “Júlio de Mesquita Filho” (UNESP), 18618-687 Botucatu, SP, Brazil

## Abstract

*Leishmania infantum* is the etiological agent of visceral leishmaniasis (VL) in the Americas with domestic dogs being its major reservoir hosts. The main VL vector is the sandfly* Lutzomyia longipalpis*, while other* Lutzomyia* species may play a role in disease transmission. Although the genetic structure of* L. infantum* populations has been widely evaluated, only a few studies have addressed this subject coupled to the genetic structure of the respective sandfly vectors. In this study, we analyzed the population structure of* L. infantum* in three major VL endemic areas in Brazil and associated it with* Lutzomyia longipalpis* geographic structure.

## 1. Introduction

Leishmaniases are parasitic diseases caused by protozoans from the genus* Leishmania*, which are transmitted by the bite of female sandflies from the family Psychodidae. The clinical manifestations of leishmaniases are particularly diverse and present different characteristics: visceral leishmaniasis (VL), the most severe one; mucocutaneous leishmaniasis, characterized as a mutilating disease; diffuse cutaneous leishmaniasis, caused by a deficient cellular immune response; and cutaneous leishmaniasis, which causes single or multiple lesions on the skin. The epidemiology of leishmaniasis is highly complex: there are 20 known species of* Leishmania* pathogenic to humans and at least 30 species of sandflies vectors. Furthermore, this disease can be designated as a zoonosis, which involves animals as the reservoir hosts or as an anthroponosis, when humans are the only source of parasites for sandflies. Leishmaniasis is widely spread in 98 countries and 3 territories, from which more than 70% are developing countries and 13 are among the least developed ones [1].

Visceral leishmaniasis can be either an anthroponosis (e.g., in the Indian subcontinent) or a zoonosis (e.g., in the Mediterranean or in the Americas), and it is characterized by chronic evolution and systemic involvement, which if untreated may result in death. In the Americas,* Leishmania infantum* is the etiological agent of the disease and Brazil accounts for over 90% of the cases in the continent [1, 2]. Domestic dogs are the proven reservoir hosts in rural and urban areas, while the role of naturally infected wild mammals (canids and marsupials) as* L. infantum* reservoir hosts is still controversial [3]. The main sandfly vector is* Lutzomyia longipalpis*, but other* Lutzomyia* species might play a role in disease transmission; for example, in Corumbá, Mato Grosso do Sul, naturally* Leishmania*-infected* Lu. cruzi* have been discovered and because there is still no evidence of* Lu. longipalpis* in this region, that sandfly is considered the main vector [4, 5].

In Brazil, VL typically occurred in rural settings, but since 1980 its incidence has been changing due to widespread urban outbreaks. The first major VL urban epidemic in the country happened in Teresina, Piauí State. Since then, epidemics occurred in Natal (Rio Grande do Norte) and São Luís (Maranhão), and the disease subsequently spread to other regions of the country. Autochthonous cases were recently described for the first time in the southernmost State of Rio Grande do Sul. The current epidemiological scenario of VL leaves no doubt regarding the severity of the situation and the unchecked geographic spread of the disease. In the 1990s, only 10% of the cases occurred outside the Northeast Region, but in 2007 the proportion reached 50% of cases. From 2006 to 2008, autochthonous transmission of VL was reported in more than 1,200 municipalities in 21 states [6].

The broad spectrum of leishmaniasis-associated symptoms, coupled with the wide diversity of vertebrate and invertebrate host species, suggests that both parasites' and hosts' genetic backgrounds determine the patterns of the disease [7]. On the other hand, clonal diversity and genetic heterogeneity, which can cause variability in parasite virulence, are quite common in* Leishmania* [8].

Several studies showed that genetic variability of* L. infantum* in Brazil is low, with restricted diversity and limited population clustering. In a recent work assessing parasite populations distributed over 18 states, three major clustered populations could be inferred using microsatellite typing. When the analysis is performed in parasites from closely related geographic regions, the overall diversity is even lower [9–11].

When we look at sandfly genetic variability, there is compelling evidence that the* Lutzomyia* population structure in Brazil is complex, with different genotypes identified depending on the geographic region assessed and also the species involved in parasite transmission [12–14].

Based on these studies, it is logical to hypothesize that the interactions of* L. infantum* genotypic variants with different hosts and vector populations may ultimately influence the transmission dynamics and severity of eventual outbreaks. Hence, assessing the genetic structure of both vector populations and parasites may help us to understand the dynamics of vector-parasite interactions and the epidemiological aspects of American visceral leishmaniasis. Here, we used PCR-RFLP of kinetoplast minicircle DNA (kDNA) to identify* L. infantum* genotypic variants from three VL endemic areas in Brazil: Teresina in Piauí State, Campo Grande in Mato Grosso do Sul State, and Bauru in São Paulo State. kDNA-RFLP analysis when compared to microsatellite genotyping has proven to be more sensitive to examine genetic data of closely related sympatric* L. infantum* strains [15]. In addition, in order to identify different haplotypes of* Lu. longipalpis* and* Lu. cruzi* sandflies from those three VL endemic areas, we used mitochondrial 12S rDNA sequencing. As a maternal inheritance, rapidly evolving, nonrecombinant and haploid molecular marker, 12S rDNA is suitable to trace genealogy and evolutionary history. To our knowledge, this is the first study that seeks to compare genetic variability of* Leishmania* infantum parasites to the genetic structure of its vectors in Brazil.

## 2. Methods

### 2.1. Ethics Statement

For insect collections in Mato Grosso do Sul State, we obtained a permanent license for collecting and transporting zoological material N° 25592-1 on behalf of Dr. Alessandra Gutierrez de Oliveira, issued by the System of Authorization and Information on Biodiversity of the Brazilian Institute of Environment and Renewable Natural Resources (Sisbio/IBAMA). For insect collections in São Paulo State and Piauí State, no specific permissions were required since the specimens were kindly provided by the Center for the Control of Endemic Diseases (SUCEN) and Federal Piauí State University, respectively. The collections were performed at private residences, whose owners personally granted permission to enter their backyards to capture the sandflies. All of these residences were located in urban areas and no endangered or protected species were collected in this study.

### 2.2. Sandfly Collections

Sandflies were captured by both manual collection and electric traps. Manual collection was performed with electric aspirators, restricting the use of a Castro catcher to locations where aspiration could not be used. The selected collection points were preassessed in order to establish the best capturing location in the peridomicile. At each selected point, modified CDC light traps were installed from 6 p.m. to 6 a.m.

The collections took place in different areas in Brazil and were performed by the respective local teams: São Paulo (SP) State, performed by Center for the Control of Endemic Diseases (SUCEN); Piauí (PI) State, performed by Piauí Federal University; and Mato Grosso do Sul (MS) State, carried out by Mato Grosso do Sul Federal University.

*Lu. cruzi* was collected in Corumbá (MS) and* Lu. longipalpis* in all other places: Campo Grande (MS), Teresina (PI), Andradina (SP), Araçatuba (SP), and Birigui (SP). All identified insects were kept in 70% ethanol until use.

### 2.3. Sandfly Genomic DNA Isolation

The field-derived sandflies were grinded with the help of a plastic pestle in 1.5 mL tubes containing 300 *μ*L of 5% Chelex® (Bio-Rad). The solution was then vortexed for 15 s, centrifuged at 11,000 g for 20 s, and incubated at 80°C for 30 min, after which the procedure was repeated. The supernatant was finally removed, transferred to another 1.5 mL microcentrifuge tube, and stored at −20°C. We had an average of 45 ng of DNA per sandfly measured with NanoDrop*™* 1000 (Thermo Scientific).

### 2.4. Sandfly Mitochondrial 12S rDNA Amplification and Sequencing

PCR amplification of the* Lutzomyia *sp. 12S rDNA mitochondrial region was performed with the primers T1B (5′-AAACTAGGATTAGATACCT-3′) and T2B (5′-AATGAGAGCGACGGGCGATG-3′), according to Beati et al. [16]. Reactions of 25 *μ*L were set up as follows: 13.7 *μ*L of ultrapure water, 2.5 *μ*L of 10x Platinum buffer (Life Technologies), 1.0 *μ*L MgCl2 (50 mM), 0.5 *μ*L dNTPs (0.1 mM), 1.0 *μ*L of each oligonucleotide (10 pmol/*μ*L), 0.3 *μ*L of Platinum Taq, (Life Technologies; 5 U/*μ*L), and 10 ng of genomic DNA. The reaction was carried out in a thermal cycler as follows: 5 cycles of 94°C for 15 s, 51°C for 30 s, and 68°C for 30 s, followed by 25 cycles of 94°C for 15 s, 53°C for 30 s, and 70°C for 30 s, and a final extension step of 70°C for 5 min. The amplified DNA fragments were UV visualized after electrophoresis on 1% agarose gel stained with ethidium bromide.

The resulting DNA fragments were purified with ExoSAP-IT kit (GE Healthcare), according to the manufacturer's protocol. The 20 *μ*L sequencing reactions consisted of 2 *μ*L of BigDye Terminator (Life Technologies), 6.0 *μ*L of BigDye Terminator 5x Sequencing Buffer (Life Technologies), 3.2 *μ*L of the primers (1 pmol/*μ*L), 4.8 *μ*L of ultrapure water, and 200 ng of DNA measured with NanoDrop 1000 (Thermo Scientific). All reactions were carried out in a thermal cycler, with 35 cycles of 95°C for 20 s, 50°C for 15 s, and 60°C for 2 min. The amplified DNA was precipitated with 80 *μ*L of 65% isopropanol, washed with 200 *μ*L of 70% ethanol, and air-dried for 5 min. Before injection, samples were resuspended in 10 *μ*L of HI-DI formamide (Life Technologies) and heated at 95°C for 3 min for DNA denaturation and immediately cooled on ice. Sample processing occurred in an ABI377 automatic sequencer.

### 2.5. Sequencing Analysis

The forward and reverse 12S rDNA sequences were manually checked for quality and the polymorphisms confirmed and then matched using the online EMBOSS GUI tool package (http://imed.med.ucm.es/cgi-bin/emboss.pl?_action=input&_app=merger). The obtained consensus sequences were aligned using Clustal X2 software. Polymorphisms in each sequence were identified and a haplotypic diversity test (Table 1) was performed with the DnaSP 5.10 software. Haplotype diagram generation was performed by TCS: phylogenetic network using statistical estimation parsimony software.

### 2.6. Parasite Samples and DNA Isolation

Parasites used in this study were collected between 2007 and 2009 (Table 2). The DNA from the promastigotes (from all cultured samples used and for the two parasite samples obtained from sandflies) was isolated with Chelex (Bio-Rad). Briefly, 1 mL aliquots of the cultures were transferred to 1.5 mL centrifuge tubes and spun down for 1 min at 10,000 g. The supernatant was discarded and the pellet resuspended in 300 *μ*L of 10% Chelex (w/v). Following, the samples were incubated for 15 min at 95°C and then centrifuged again for 1 min at 10,000 g. The supernatant containing the DNA was then carefully recovered and stored in a new tube at −20°C. For the two parasite samples isolated from sandflies, the whole insect was grinded in 300 *μ*L of 10% Chelex with the help of a motorized tissue grinder, following the same steps above. We had an average of 200 ng of DNA per culture sampled and 20 ng per sample for the two sandfly-derived parasites measured with NanoDrop 1000 (Thermo Scientific).

The DNA of* L. infantum* amastigotes was extracted following two different approaches. For dog bone marrow aspirates we used the Illustra Blood GenomicPrep Mini Spin kit (GE Healthcare) according to the manufacturer's recommendations. For slide-fixed human bone marrow aspirates we used the same protocol after scraping the contents of each slide into a 1.5 mL tube, as previously described [17]. We had an average of 100 ng per dog bone marrow sample and 25 ng of DNA per slide measured with NanoDrop 1000 (Thermo Scientific).

### 2.7. PCR-RFLP of Kinetoplast DNA (kDNA) and RFLP Analysis

We had initially started our analysis using a panel of 7 microsatellite markers (Li22-35, Li23-41, Li45-24, Li71-33, Lm2TG, Lm4TA, and TubCA) [10]. However, only one marker (Li45-24) was polymorphic and, due to its low variability, only two alleles could be identified. For this reason, we decided to perform only PCR-RFLP of kinetoplast DNA (kDNA) and RFLP analysis.

For the analysis of the kinetoplast minicircle DNA, 157* L. infantum* isolates were used (Table 2): 98 cultured samples initially isolated from human patients by sternal bone marrow aspiration (44 from Teresina and 54 from Campo Grande), 42 samples from dog bone marrow aspirates from Teresina, 2 samples from sandflies blood-fed on* L. infantum*-infected dogs from this same study in Teresina, and 15 slide-derived samples originated from bone marrow aspirates of human patients in Bauru, São Paulo State. PCR reactions were performed with primers LINR4 and LIN19 [18] and generated a 720 bp amplicon, which covers almost the entire minicircle. The 50 *μ*L reactions contained 1 mM MgCl2, 10 mM Tris-HCl (pH 8.3), 0.3 pmol of each oligonucleotide, 0.1 mM dNTPs, 1 unit of Taq polymerase (GE Healthcare), and 5 *μ*L of sample DNA. The amplification conditions were as follows: 3 min at 94°C, 33 cycles of 30 s at 95°C, 30 s at 58°C, and 1 min at 72°C, followed by a final extension step of 10 minutes at 72°C. The PCR products were then precipitated with ethanol, resuspended in water and digested with the restriction enzymes RsaI and HpaII (Promega) as previously described [15]. Approximately 1 *μ*g of each PCR product was used per digestion in order to ensure that all reactions had the same initial amount of DNA. Since the products smaller than 100 bp can be confused with primer dimers and the ones larger than 700 bp can be misidentified as undigested products, only the fragments within this range were used in our RFLP analysis.

Data analysis was performed using R software environment. A binary matrix was constructed based on the profile of fragments generated by each digestion, where 1 represents the presence of a fragment and 0 represents its absence. This matrix was converted into a similarity matrix using the package “proxy” and used for cluster analysis. After, *K*-means partitioning method was used to infer the number of clusters using the package “*k*-means” and Agglomerative Hierarchical Clustering dendrogram was built using the binary distance method and ward cluster method with the package “hclust”.

## 3. Results

### 3.1. Sandflies Genetic Analysis

**Figure 1:**
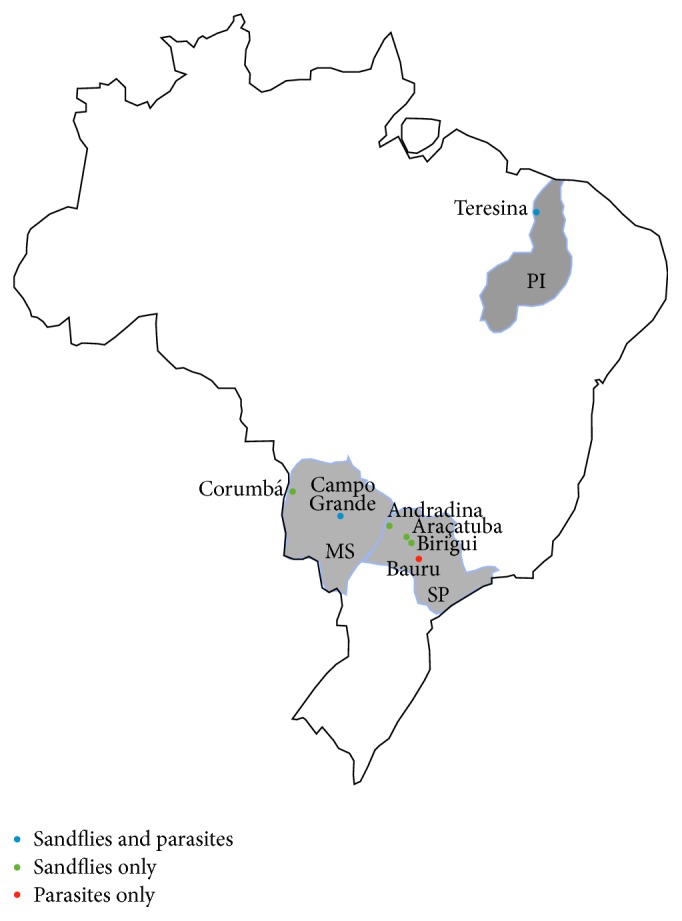
Map of Brazil, with emphasis on the states of Mato Grosso do Sul (MS), São Paulo (SP), and Piauí (PI). The position of each studied locality in the states where samples were collected is depicted.

**Figure 2:**
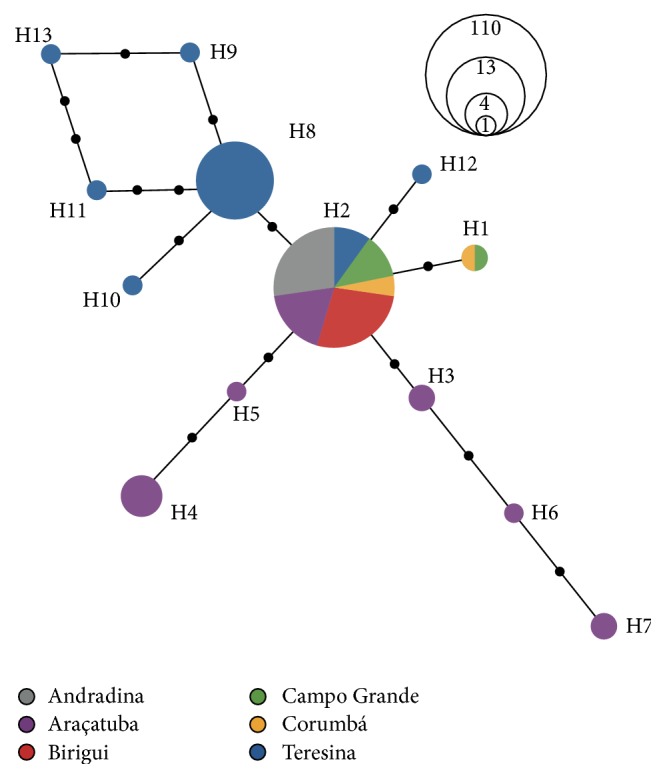
The diagram of 12S mitochondrial haplotypes generated for* Lutzomyia *sp. Haplotypes found after the analysis of a 263 bp fragment of 12S mitochondrial rRNA. The diameter of the circles is related to the numbers of individuals found with the same haplotype. The connections between haplotypes are of the same size in relation to the center of each circle. The black dots represent the number of steps (SNPs) between the haplotypes.

DNA was extracted from a total of 140 individuals as follows: 30 individuals from Andradina (SP), Araçatuba (SP), and Birigui (SP); 29 individuals from Teresina (PI); 14 individuals from Campo Grande (MS); 7 individuals from Corumbá (MS), classified morphologically as* Lu*.* cruzi* (Figure 1). PCR reactions generated a mitochondrial 12S ribosomal DNA fragment of approximately 360 bp, as previously described [19], which was then partially sequenced (263 bp). Sequences were screened for significant polymorphisms, and 10 variable sites were found (Table 3). When polymorphisms were assessed with DnaSP 5.10 program, 13 haplotypes were generated: six haplotypes (H8, H9, H10, H11, H12, and H13) containing only individuals from Teresina (PI); five haplotypes (H3, H4, H5, H6, and H7) containing only Araçatuba individuals (SP); one haplotype (H1) containing one individual from Corumbá and one individual from Campo Grande (MS); and one haplotype (H2) covering most of the sequences (111 individuals). Data are represented in a diagram of haplotypes (Figure 2). The haplotypic diversity test showed that Teresina presented the highest diversity (0.672), followed by Araçatuba (0.545), Corumbá (0.286), and Campo Grande (0.143). Andradina and Birigui presented no haplotypic diversity at all (Table 1).

### 3.2. Parasites RFLP Analysis

**Figure 3:**
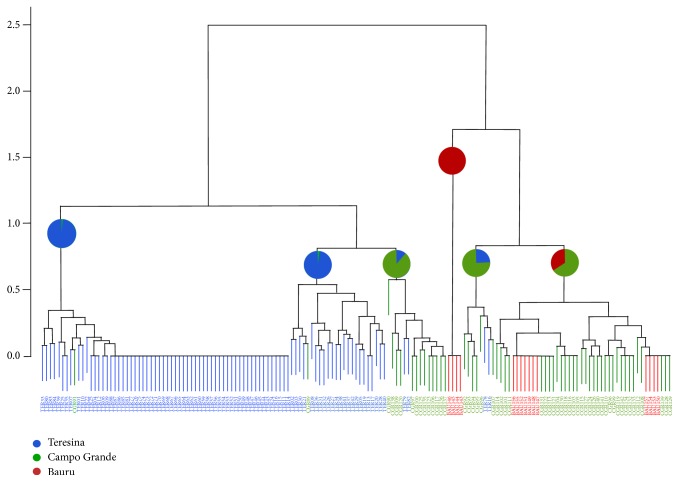
Cluster analysis generated from PCR-RFLP data for* Leishmania infantum*. Hierarchical Agglomerative Clustering for 157 samples of* Leishmania infantum* parasites assessed in the study. *K*-means partitioning identified six major clusters, which are depicted with pie charts containing the proportions of parasites from each geographic area assessed.

The kDNA fragments of interest were successfully amplified from the LinR4 and Lin19 oligos used in this study. RFLP analysis of kinetoplast minicircles DNA was also efficient in detecting restriction patterns between different samples. From the 157 tested samples, we could observe 55 unique genotypes in the cluster analysis dendrogram illustrated in Figure 3. *K*-means partitioning identified 6 major clusters; there was a clear distinction between samples from Teresina, which grouped in two almost exclusive clusters, and all other samples; an exclusive Bauru cluster was also found. Two clusters presented with Teresina and Campo Grande samples, and one cluster presented with Bauru and Campo Grande samples. It is noteworthy that Campo Grande is distributed over 3 major clusters, one that groups together with one Teresina major cluster and the other two that are closer to Bauru clusters in a separate branch of the dendrogram. There was no clustering differentiation related to the years of collection.

## 4. Discussion

During the past 20 years, the epidemiology of VL has been constantly changing due to a continuous urbanization process, an increasing incidence of HIV/*Leishmania* coinfections, and syringe sharing by intravenous drug users [20] and the identification of novel* L. infantum* mammalian hosts/reservoirs [21]. This highlights the necessity of molecularly tracking the geographic distribution of different parasite and vector populations in order to enhance the knowledge on basic epidemiological aspects of the disease, such as its natural history and transmission.

Several molecular approaches have been used in the characterization of genetic variants in the genus* Leishmania*: amplified polymorphic DNA (RAPD) markers [22], analysis by size polymorphism of restriction fragments (RFLP) of the ITS regions ribosomal DNA [23], and kinetoplast DNA [24]; analysis confirmed sequence amplified regions [25]; and analysis of regions of DNA with microsatellite markers [19, 26–29]. We then decided to proceed with PCR-RFLP analysis of minicircle kDNA because it has a higher resolving power when applied to population genetics studies involving either genetically or geographically closely related strains [24, 30, 31]. Our data revealed a clear distinction between samples from Teresina, which grouped in two almost exclusive clusters, and all other samples; an exclusive Bauru cluster was also found. Two clusters presented with Teresina and Campo Grande samples, and one cluster presented with Bauru and Campo Grande samples. These results allowed us to draw a relationship between genetic distance and geographic origin. Interestingly, geographic origin related to diverse genetic background was also found for* L. infantum* parasites in Brazil in the study performed by Segatto et al. [10].

Our data is partially in accordance with a previous microsatellite based genotyping study performed with parasite populations from all 5 Brazilian regions. In the study, three well-defined populations could be identified; one that was present mostly in Northeast region, (including Piauí State that was sampled in our study) and the other two present in Midwest region (including Mato Grosso and Mato Grosso do Sul States that were sampled in our study). On the other hand, parasites typed in Southeast region (including São Paulo State that was sampled in our study) are closely related to northeastern parasites while in our study they are closely related to Midwestern parasites [9]. Our findings corroborate the use of this technique in* Leishmania* genotyping studies and reinforce the idea that in some cases, especially when analyzing strains of very close geographical origin, it is the only molecular marker capable of producing detectable patterns of polymorphism [24, 32].

All these genotyping studies on* L. infantum* suggest that the nuclear genomic variability of this species is likely to be low. Our hypothesis is that the kinetoplast genome can serve as a source of genetic variability for these parasites. The kDNA minicircles are essential for the function of the trypanosomatid's mitochondrial genes, as minicircles code for guide RNAs, which play an essential role in editing messenger RNA (mRNA) from the maxicircles that contain genes for essential mitochondrial proteins [33]. Therefore, this DNA is more prone to a rapid response to diverse ambient conditions and stress situations, and parasite fitness conferring different selective advantages might depend on which minicircle classes prevail in different* Leishmania* strains.

A similar phenomenon, known as transkinetoplastidy, has been described in* Leishmania* and is responsible for changes in minicircles classes when the parasites are challenged with increasing concentrations of drugs that are normally lethal. This will in turn cause a dramatic change in the abundance of certain minicircles classes, which during transkinetoplastidy will be increased or reduced and replaced by a previously less frequent class [34].

When we look at sandfly genetic analysis we can clearly observe a main haplotype (H2) comprising all individuals from Andradina and Birigui, 13 out of 14 individuals from Campo Grande, 6 out of 7 individuals from Corumbá, 20 out of 30 individuals from Araçatuba, and 11 out 30 individuals from Teresina. There is also a major haplotype (H8) comprising only individuals from Teresina (13 out of 29) and minor haplotypes from Araçatuba. From the 12S rDNA sequencing data, it was not possible to differentiate* Lu. longipalpis* from* Lu. cruzi* (Corumbá) since there was no haplotype clustering among Corumbá sandflies. This may suggest that the process of speciation is recent or still occurring. A microsatellite based study assessing the genetic variability of* Lu. longipalpis* and* Lu. cruzi* populations in Mato Grosso do Sul State showed evidence of introgression and limited gene flow between the two species, corroborating our findings [12].

In general, we can summarize the data obtained from haplotyping as follows: a major haplotype composed of 111 individuals (comprising 89% of SP, 90% of MS, and 38% of PI individuals); a main haplotype composed of 13 individuals exclusively from Teresina and giving rise to other 4 Teresina exclusive haplotypes (62% of individuals from Teresina with exclusive haplotypes); minor haplotypes comprising only individuals from SP (11% total) and from the same locality (Araçatuba).

When we compare data from parasite genotyping with sandfly 12S rDNA sequencing, the correlation of the two datasets is remarkable. Both show most samples from PI clearly separated from the MS and SP ones which are in turn much more related to each other when compared to PI that presented the highest haplotypic diversity (Table 1). The exception comes from the minor vector haplotypes only found in Araçatuba samples. Araçatuba represents an important landmark in the natural history of VL in SP given the fact that the first VL outbreak registered in the state occurred in this location [35, 36]. This could be a possible explanation to its greater number of unique haplotypes as one can assume that coevolution between parasites and vectors happens for a longer time in this area; this is supported by the high haplotypic diversity found for this population (Table 1). Taken together, these data corroborate that the sandfly vector probably plays an important role in shaping the genetic structure of* L. infantum* in Brazil as described by Ferreira et al. [9].

This work presents new insights towards the understanding of the population structure of* L. infantum* and* Lu. longipalpis* from VL endemic areas in Brazil. Further analyses will be needed to elucidate how different vector populations shape the genetic variability of* L. infantum*.

## 5. Conclusions

Taken together, our data indicate that the sandfly vector might play a role in selecting specific parasite strains at a regional level and therefore contributing to the genetic structure of* L. infantum* in Brazil. Assessing the genetic structure of both vector and parasite populations may help us to understand the evolution process surrounding vector-parasite interactions and shed light on a fundamental aspect of the ecoepidemiology of American visceral leishmaniasis.

## Figures and Tables

**Table 1:** Haplotype diversity analysis of the six sandfly populations assessed.

Populations sampled	Number of individuals sampled (*N*)	Number of haplotypes	Haplotype diversity (Hd)	Variance of haplotype diversity	Standard deviation of haplotype diversity
Andradina	30	1	0	0	0
Araçatuba	30	6	0.545	0.01027	0.101
Birigui	30	1	0	0	0
Campo Grande	14	2	0.143	0.01412	0.119
Corumbá	7	2	0.286	0.03856	0.196
Teresina	29	7	0.672	0.00346	0.059

**Table 2:** Parasite samples genotyped in the study.

Laboratory code	WHO code	Life stage	Type of sample	Host	Year of isolation	Location
TER1	MCAN/BR/2007/TER1	Amastigotes	Fresh blood marrow aspirates	Dog	2007	Teresina, PI
TER2	MCAN/BR/2007/TER2	Amastigotes	Fresh blood marrow aspirates	Dog	2007	Teresina, PI
TER3	MCAN/BR/2007/TER3	Amastigotes	Fresh blood marrow aspirates	Dog	2007	Teresina, PI
TER4	MCAN/BR/2007/TER4	Amastigotes	Fresh blood marrow aspirates	Dog	2007	Teresina, PI
TER5	MCAN/BR/2007/TER5	Amastigotes	Fresh blood marrow aspirates	Dog	2007	Teresina, PI
TER6	MCAN/BR/2007/TER6	Amastigotes	Fresh blood marrow aspirates	Dog	2007	Teresina, PI
TER7	MCAN/BR/2007/TER7	Amastigotes	Fresh blood marrow aspirates	Dog	2007	Teresina, PI
TER8	MCAN/BR/2007/TER8	Amastigotes	Fresh blood marrow aspirates	Dog	2007	Teresina, PI
TER9	MCAN/BR/2008/TER9	Amastigotes	Fresh blood marrow aspirates	Dog	2008	Teresina, PI
TER10	MCAN/BR/2008/TER10	Amastigotes	Fresh blood marrow aspirates	Dog	2008	Teresina, PI
TER11	MCAN/BR/2008/TER11	Amastigotes	Fresh blood marrow aspirates	Dog	2008	Teresina, PI
TER12	MCAN/BR/2008/TER12	Amastigotes	Fresh blood marrow aspirates	Dog	2008	Teresina, PI
TER13	MCAN/BR/2008/TER13	Amastigotes	Fresh blood marrow aspirates	Dog	2008	Teresina, PI
TER14	MCAN/BR/2008/TER14	Amastigotes	Fresh blood marrow aspirates	Dog	2008	Teresina, PI
TER15	MCAN/BR/2008/TER15	Amastigotes	Fresh blood marrow aspirates	Dog	2008	Teresina, PI
TER16	MCAN/BR/2008/TER16	Amastigotes	Fresh blood marrow aspirates	Dog	2008	Teresina, PI
TER17	MCAN/BR/2008/TER17	Amastigotes	Fresh blood marrow aspirates	Dog	2008	Teresina, PI
TER18	MCAN/BR/2008/TER18	Amastigotes	Fresh blood marrow aspirates	Dog	2008	Teresina, PI
TER19	MCAN/BR/2008/TER19	Amastigotes	Fresh blood marrow aspirates	Dog	2008	Teresina, PI
TER20	MCAN/BR/2008/TER20	Amastigotes	Fresh blood marrow aspirates	Dog	2008	Teresina, PI
TER21	MCAN/BR/2009/TER21	Amastigotes	Fresh blood marrow aspirates	Dog	2009	Teresina, PI
TER22	MCAN/BR/2009/TER22	Amastigotes	Fresh blood marrow aspirates	Dog	2009	Teresina, PI
TER23	MCAN/BR/2009/TER23	Amastigotes	Fresh blood marrow aspirates	Dog	2009	Teresina, PI
TER24	MCAN/BR/2009/TER24	Amastigotes	Fresh blood marrow aspirates	Dog	2009	Teresina, PI
TER25	MCAN/BR/2009/TER25	Amastigotes	Fresh blood marrow aspirates	Dog	2009	Teresina, PI
TER26	MCAN/BR/2009/TER26	Amastigotes	Fresh blood marrow aspirates	Dog	2009	Teresina, PI
TER27	MCAN/BR/2009/TER27	Amastigotes	Fresh blood marrow aspirates	Dog	2009	Teresina, PI
TER28	MCAN/BR/2009/TER28	Amastigotes	Fresh blood marrow aspirates	Dog	2009	Teresina, PI
TER29	MCAN/BR/2009/TER29	Amastigotes	Fresh blood marrow aspirates	Dog	2009	Teresina, PI
TER30	MCAN/BR/2009/TER30	Amastigotes	Fresh blood marrow aspirates	Dog	2009	Teresina, PI
TER31	MCAN/BR/2009/TER31	Amastigotes	Fresh blood marrow aspirates	Dog	2009	Teresina, PI
TER32	MCAN/BR/2009/TER32	Amastigotes	Fresh blood marrow aspirates	Dog	2009	Teresina, PI
TER33	MCAN/BR/2009/TER33	Amastigotes	Fresh blood marrow aspirates	Dog	2009	Teresina, PI
TER34	MCAN/BR/2009/TER34	Amastigotes	Fresh blood marrow aspirates	Dog	2009	Teresina, PI
TER35	MCAN/BR/2009/TER35	Amastigotes	Fresh blood marrow aspirates	Dog	2009	Teresina, PI
TER36	MCAN/BR/2009/TER36	Amastigotes	Fresh blood marrow aspirates	Dog	2009	Teresina, PI
TER37	MCAN/BR/2009/TER37	Amastigotes	Fresh blood marrow aspirates	Dog	2009	Teresina, PI
TER38	MCAN/BR/2009/TER38	Amastigotes	Fresh blood marrow aspirates	Dog	2009	Teresina, PI
TER39	MCAN/BR/2009/TER39	Amastigotes	Fresh blood marrow aspirates	Dog	2009	Teresina, PI
TER40	MCAN/BR/2009/TER40	Amastigotes	Fresh blood marrow aspirates	Dog	2009	Teresina, PI
TER41	MCAN/BR/2009/TER41	Amastigotes	Fresh blood marrow aspirates	Dog	2009	Teresina, PI
TER42	MCAN/BR/2009/TER42	Amastigotes	Fresh blood marrow aspirates	Dog	2009	Teresina, PI
TER43	ILON/BR/2009/TER43	Promastigotes	Cultured parasites	Sandfly	2009	Teresina, PI
TER44	ILON/BR/2009/TER44	Promastigotes	Cultured parasites	Sandfly	2009	Teresina, PI
TER45	MHOM/BR/2009/TER45	Promastigotes	Cultured parasites	Human	2009	Teresina, PI
TER46	MHOM/BR/2008/TER46	Promastigotes	Cultured parasites	Human	2008	Teresina, PI
TER47	MHOM/BR/2008/TER47	Promastigotes	Cultured parasites	Human	2008	Teresina, PI
TER48	MHOM/BR/2008/TER48	Promastigotes	Cultured parasites	Human	2008	Teresina, PI
TER49	MHOM/BR/2008/TER49	Promastigotes	Cultured parasites	Human	2008	Teresina, PI
TER50	MHOM/BR/2008/TER50	Promastigotes	Cultured parasites	Human	2008	Teresina, PI
TER51	MHOM/BR/2008/TER51	Promastigotes	Cultured parasites	Human	2008	Teresina, PI
TER52	MHOM/BR/2007/TER52	Promastigotes	Cultured parasites	Human	2007	Teresina, PI
TER53	MHOM/BR/2007/TER53	Promastigotes	Cultured parasites	Human	2007	Teresina, PI
TER54	MHOM/BR/2007/TER54	Promastigotes	Cultured parasites	Human	2007	Teresina, PI
TER55	MHOM/BR/2007/TER55	Promastigotes	Cultured parasites	Human	2007	Teresina, PI
TER56	MHOM/BR/2007/TER56	Promastigotes	Cultured parasites	Human	2007	Teresina, PI
TER57	MHOM/BR/2007/TER57	Promastigotes	Cultured parasites	Human	2007	Teresina, PI
TER58	MHOM/BR/2007/TER58	Promastigotes	Cultured parasites	Human	2007	Teresina, PI
TER59	MHOM/BR/2007/TER59	Promastigotes	Cultured parasites	Human	2007	Teresina, PI
TER60	MHOM/BR/2007/TER60	Promastigotes	Cultured parasites	Human	2007	Teresina, PI
TER61	MHOM/BR/2007/TER61	Promastigotes	Cultured parasites	Human	2007	Teresina, PI
TER62	MHOM/BR/2007/TER62	Promastigotes	Cultured parasites	Human	2007	Teresina, PI
TER63	MHOM/BR/2007/TER63	Promastigotes	Cultured parasites	Human	2007	Teresina, PI
TER64	MHOM/BR/2007/TER64	Promastigotes	Cultured parasites	Human	2007	Teresina, PI
TER65	MHOM/BR/2007/TER65	Promastigotes	Cultured parasites	Human	2007	Teresina, PI
TER66	MHOM/BR/2009/TER66	Promastigotes	Cultured parasites	Human	2009	Teresina, PI
TER67	MHOM/BR/2009/TER67	Promastigotes	Cultured parasites	Human	2009	Teresina, PI
TER68	MHOM/BR/2009/TER68	Promastigotes	Cultured parasites	Human	2009	Teresina, PI
TER69	MHOM/BR/2009/TER69	Promastigotes	Cultured parasites	Human	2009	Teresina, PI
TER70	MHOM/BR/2009/TER70	Promastigotes	Cultured parasites	Human	2009	Teresina, PI
TER71	MHOM/BR/2009/TER71	Promastigotes	Cultured parasites	Human	2009	Teresina, PI
TER72	MHOM/BR/2009/TER72	Promastigotes	Cultured parasites	Human	2009	Teresina, PI
TER73	MHOM/BR/2009/TER73	Promastigotes	Cultured parasites	Human	2009	Teresina, PI
TER74	MHOM/BR/2009/TER74	Promastigotes	Cultured parasites	Human	2009	Teresina, PI
TER75	MHOM/BR/2009/TER75	Promastigotes	Cultured parasites	Human	2009	Teresina, PI
TER76	MHOM/BR/2009/TER76	Promastigotes	Cultured parasites	Human	2009	Teresina, PI
TER77	MHOM/BR/2009/TER77	Promastigotes	Cultured parasites	Human	2009	Teresina, PI
TER78	MHOM/BR/2009/TER78	Promastigotes	Cultured parasites	Human	2009	Teresina, PI
TER79	MHOM/BR/2009/TER79	Promastigotes	Cultured parasites	Human	2009	Teresina, PI
TER80	MHOM/BR/2009/TER80	Promastigotes	Cultured parasites	Human	2009	Teresina, PI
TER81	MHOM/BR/2008/TER81	Promastigotes	Cultured parasites	Human	2008	Teresina, PI
TER82	MHOM/BR/2008/TER82	Promastigotes	Cultured parasites	Human	2008	Teresina, PI
TER83	MHOM/BR/2008/TER83	Promastigotes	Cultured parasites	Human	2008	Teresina, PI
TER84	MHOM/BR/2008/TER84	Promastigotes	Cultured parasites	Human	2008	Teresina, PI
TER85	MHOM/BR/2008/TER85	Promastigotes	Cultured parasites	Human	2008	Teresina, PI
TER86	MHOM/BR/2008/TER86	Promastigotes	Cultured parasites	Human	2008	Teresina, PI
TER87	MHOM/BR/2008/TER87	Promastigotes	Cultured parasites	Human	2008	Teresina, PI
TER88	MHOM/BR/2009/TER88	Promastigotes	Cultured parasites	Human	2009	Teresina, PI
CGR89	MHOM/BR/2009/CGR89	Promastigotes	Cultured parasites	Human	2009	Campo Grande, MS
CGR90	MHOM/BR/2009/CGR90	Promastigotes	Cultured parasites	Human	2009	Campo Grande, MS
CGR91	MHOM/BR/2009/CGR91	Promastigotes	Cultured parasites	Human	2009	Campo Grande, MS
CGR92	MHOM/BR/2009/CGR92	Promastigotes	Cultured parasites	Human	2009	Campo Grande, MS
CGR93	MHOM/BR/2009/CGR93	Promastigotes	Cultured parasites	Human	2009	Campo Grande, MS
CGR94	MHOM/BR/2009/CGR94	Promastigotes	Cultured parasites	Human	2009	Campo Grande, MS
CGR95	MHOM/BR/2009/CGR95	Promastigotes	Cultured parasites	Human	2009	Campo Grande, MS
CGR96	MHOM/BR/2009/CGR96	Promastigotes	Cultured parasites	Human	2009	Campo Grande, MS
CGR97	MHOM/BR/2009/CGR97	Promastigotes	Cultured parasites	Human	2009	Campo Grande, MS
CGR98	MHOM/BR/2009/CGR98	Promastigotes	Cultured parasites	Human	2009	Campo Grande, MS
CGR99	MHOM/BR/2009/CGR99	Promastigotes	Cultured parasites	Human	2009	Campo Grande, MS
CGR100	MHOM/BR/2009/CGR100	Promastigotes	Cultured parasites	Human	2009	Campo Grande, MS
CGR101	MHOM/BR/2009/CGR101	Promastigotes	Cultured parasites	Human	2009	Campo Grande, MS
CGR102	MHOM/BR/2009/CGR102	Promastigotes	Cultured parasites	Human	2009	Campo Grande, MS
CGR103	MHOM/BR/2009/CGR103	Promastigotes	Cultured parasites	Human	2009	Campo Grande, MS
CGR104	MHOM/BR/2009/CGR104	Promastigotes	Cultured parasites	Human	2009	Campo Grande, MS
CGR105	MHOM/BR/2009/CGR105	Promastigotes	Cultured parasites	Human	2009	Campo Grande, MS
CGR106	MHOM/BR/2009/CGR106	Promastigotes	Cultured parasites	Human	2009	Campo Grande, MS
CGR107	MHOM/BR/2009/CGR107	Promastigotes	Cultured parasites	Human	2009	Campo Grande, MS
CGR108	MHOM/BR/2009/CGR108	Promastigotes	Cultured parasites	Human	2009	Campo Grande, MS
CGR109	MHOM/BR/2009/CGR109	Promastigotes	Cultured parasites	Human	2009	Campo Grande, MS
CGR110	MHOM/BR/2009/CGR110	Promastigotes	Cultured parasites	Human	2009	Campo Grande, MS
CGR111	MHOM/BR/2008/CGR111	Promastigotes	Cultured parasites	Human	2008	Campo Grande, MS
CGR112	MHOM/BR/2008/CGR112	Promastigotes	Cultured parasites	Human	2008	Campo Grande, MS
CGR113	MHOM/BR/2008/CGR113	Promastigotes	Cultured parasites	Human	2008	Campo Grande, MS
CGR114	MHOM/BR/2008/CGR114	Promastigotes	Cultured parasites	Human	2008	Campo Grande, MS
CGR115	MHOM/BR/2008/CGR115	Promastigotes	Cultured parasites	Human	2008	Campo Grande, MS
CGR116	MHOM/BR/2008/CGR116	Promastigotes	Cultured parasites	Human	2008	Campo Grande, MS
CGR117	MHOM/BR/2008/CGR117	Promastigotes	Cultured parasites	Human	2008	Campo Grande, MS
CGR118	MHOM/BR/2008/CGR118	Promastigotes	Cultured parasites	Human	2008	Campo Grande, MS
CGR119	MHOM/BR/2008/CGR119	Promastigotes	Cultured parasites	Human	2008	Campo Grande, MS
CGR120	MHOM/BR/2008/CGR120	Promastigotes	Cultured parasites	Human	2008	Campo Grande, MS
CGR121	MHOM/BR/2008/CGR121	Promastigotes	Cultured parasites	Human	2008	Campo Grande, MS
CGR122	MHOM/BR/2008/CGR122	Promastigotes	Cultured parasites	Human	2008	Campo Grande, MS
CGR123	MHOM/BR/2008/CGR123	Promastigotes	Cultured parasites	Human	2008	Campo Grande, MS
CGR124	MHOM/BR/2008/CGR124	Promastigotes	Cultured parasites	Human	2008	Campo Grande, MS
CGR125	MHOM/BR/2008/CGR125	Promastigotes	Cultured parasites	Human	2008	Campo Grande, MS
CGR126	MHOM/BR/2008/CGR126	Promastigotes	Cultured parasites	Human	2008	Campo Grande, MS
CGR127	MHOM/BR/2008/CGR127	Promastigotes	Cultured parasites	Human	2008	Campo Grande, MS
CGR128	MHOM/BR/2008/CGR128	Promastigotes	Cultured parasites	Human	2008	Campo Grande, MS
CGR129	MHOM/BR/2008/CGR129	Promastigotes	Cultured parasites	Human	2008	Campo Grande, MS
CGR130	MHOM/BR/2009/CGR130	Promastigotes	Cultured parasites	Human	2009	Campo Grande, MS
CGR131	MHOM/BR/2009/CGR131	Promastigotes	Cultured parasites	Human	2009	Campo Grande, MS
CGR132	MHOM/BR/2009/CGR132	Promastigotes	Cultured parasites	Human	2009	Campo Grande, MS
CGR133	MHOM/BR/2009/CGR133	Promastigotes	Cultured parasites	Human	2009	Campo Grande, MS
CGR134	MHOM/BR/2009/CGR134	Promastigotes	Cultured parasites	Human	2009	Campo Grande, MS
CGR135	MHOM/BR/2009/CGR135	Promastigotes	Cultured parasites	Human	2009	Campo Grande, MS
CGR136	MHOM/BR/2007/CGR136	Promastigotes	Cultured parasites	Human	2007	Campo Grande, MS
CGR137	MHOM/BR/2007/CGR137	Promastigotes	Cultured parasites	Human	2007	Campo Grande, MS
CGR138	MHOM/BR/2007/CGR138	Promastigotes	Cultured parasites	Human	2007	Campo Grande, MS
CGR139	MHOM/BR/2007/CGR139	Promastigotes	Cultured parasites	Human	2007	Campo Grande, MS
CGR140	MHOM/BR/2007/CGR140	Promastigotes	Cultured parasites	Human	2007	Campo Grande, MS
CGR141	MHOM/BR/2007/CGR141	Promastigotes	Cultured parasites	Human	2007	Campo Grande, MS
CGR142	MHOM/BR/2007/CGR142	Promastigotes	Cultured parasites	Human	2007	Campo Grande, MS
BAU143	MHOM/BR/2007/BAU143	Amastigotes	Bone marrow aspirates slides	Human	2007	Bauru, SP
BAU144	MHOM/BR/2007/BAU144	Amastigotes	Bone marrow aspirates slides	Human	2007	Bauru, SP
BAU145	MHOM/BR/2007/BAU145	Amastigotes	Bone marrow aspirates slides	Human	2007	Bauru, SP
BAU146	MHOM/BR/2008/BAU146	Amastigotes	Bone marrow aspirates slides	Human	2008	Bauru, SP
BAU147	MHOM/BR/2008/BAU147	Amastigotes	Bone marrow aspirates slides	Human	2008	Bauru, SP
BAU148	MHOM/BR/2008/BAU148	Amastigotes	Bone marrow aspirates slides	Human	2008	Bauru, SP
BAU149	MHOM/BR/2008/BAU149	Amastigotes	Bone marrow aspirates slides	Human	2008	Bauru, SP
BAU150	MHOM/BR/2008/BAU150	Amastigotes	Bone marrow aspirates slides	Human	2009	Bauru, SP
BAU151	MHOM/BR/2007/BAU151	Amastigotes	Bone marrow aspirates slides	Human	2007	Bauru, SP
BAU152	MHOM/BR/2009/BAU152	Amastigotes	Bone marrow aspirates slides	Human	2009	Bauru, SP
BAU153	MHOM/BR/2009/BAU153	Amastigotes	Bone marrow aspirates slides	Human	2009	Bauru, SP
BAU154	MHOM/BR/2009/BAU154	Amastigotes	Bone marrow aspirates slides	Human	2009	Bauru, SP
BAU155	MHOM/BR/2009/BAU155	Amastigotes	Bone marrow aspirates slides	Human	2009	Bauru, SP
BAU156	MHOM/BR/2009/BAU156	Amastigotes	Bone marrow aspirates slides	Human	2009	Bauru, SP
BAU157	MHOM/BR/2009/BAU157	Amastigotes	Bone marrow aspirates slides	Human	2009	Bauru, SP

**Table 3:** Variable sites per haplotype of 12S mitochondrial DNA in *Lutzomyia *sp.

Haplotypes	SNPs									
H1	C	T	C	C	C	T	G	T	A	T
H2	·	C	·	·	·	·	·	·	·	·
H3	T	C	·	·	·	·	·	·	·	·
H4	·	C	·	·	·	G	·	·	·	G
H5	·	C	·	·	·	G	·	·	·	·
H6	T	C	·	·	·	·	·	·	·	G
H7	T	C	·	T	·	·	·	·	·	G
H8	·	C	·	·	·	·	·	C	·	·
H9	·	C	·	·	·	·	·	C	G	·
H10	·	C	·	·	T	·	·	C	·	·
H11	·	C	T	T	·	·	·	C	·	·
H12	·	C	·	·	·	·	A	·	·	·
H13	·	C	·	T	T	·	·	C	·	·
SNPs position^a^	36	71	80	84	107	178	194	243	244	257

^a^SNPs positions are given in relation to the beginning of 12S rDNA sequence deposited as KF485516 in GenBank.
